# Relationship Orientation, Justice Perception, and Opportunistic Behavior in PPP Projects: An Empirical Study From China

**DOI:** 10.3389/fpsyg.2021.635447

**Published:** 2021-04-01

**Authors:** Guoli Feng, Shengyue Hao, Xiaoguang Li

**Affiliations:** ^1^School of Economics and Management, Beijing Jiaotong University, Beijing, China; ^2^School of Economics, Peking University, Beijing, China; ^3^China International Economic Consultants, Beijing, China

**Keywords:** relationship orientation, opportunistic behavior, justice perception, public-private partnership, SEM

## Abstract

An equal and high-quality partnership between public and private sectors is essential to the sustainable development of public–private partnership (PPP) projects. However, in the special social circumstance in China, the public sector has a strong voice in PPP projects. According to the existing research on PPP project failure, the government's dishonest performance and negative cooperative attitude and the private sector's speculative behavior of concealing information will lead to termination or even failure of project. The attitude and behavior that reflect the relationship orientation of public sector may determine whether the private sector adopts an opportunistic behavior. However, few studies have revealed the mechanism of relationship orientation on opportunism in PPP projects. This paper proposes the connotation of the public sector's relationship orientation and designs a measurement scale from three aspects: emotional relationship orientation, instrumental relationship orientation, and rent-seeking relationship orientation. Based on the data from large construction enterprises, financial institutions and investors, and scholars with practical experience in PPP projects, this paper explores the mechanism of the public sector's relationship orientation on the private sector's justice perception and opportunistic behavior by using the structural equation model (SEM). The results show that the public sector's relationship orientation significantly affects the formation and development of the private sector's justice perception and opportunistic behavior, justice perception plays a mediating role in the process of relationship orientation acting on opportunistic behavior, and the instrumental relationship orientation is more conducive to reducing the opportunistic behavior. The results provide new ideas for changing the public sector's concept and attitude and regulating behavior in PPP projects.

## Introduction

Public–private partnership (PPP) is a “whole process” cooperative relationship between government and social capital to provide public products or services (Xueguo et al., 2020). Since 2013, PPP projects have developed rapidly in China. As of December 2020, there are 9,930 projects in China, with a total investment of 152,781 billion yuan. The PPP has gradually become the main mechanism for the supply of public products and services in China, which has effectively promoted new urbanization and infrastructure construction, and accelerated the pace of supply-side structural reform.

In an ideal PPP project partnership, the public sector and the private sector should have an equal, friendly and sustainable cooperative relationship. However, in the practice of PPP in China, many local public departments only regard the PPP model as a financing method, and do not pay attention to the cultivation of partnership, which can easily lead to the confrontational attitude of the private sector and then lead to opportunistic behavior (Yilin et al., 2020). In this way, it not only is unable to alleviate the local financial pressure but also failed to improve the supply efficiency and quality of infrastructure and public services, which seriously deviated from the original intention of the implementation of PPP, resulting in frequent cases of project failure (Zhang and Tariq, 2020).

In the context of China, the public sector is not only responsible for the supply of local public facilities and services but also the maker of the PPP policy system. In cooperation with the private sector, the public sector is often in a relatively strong position and has absolute decision-making power and autonomy. The attitude and behavior of the public sector have a strong impact on the private sector. This influence is not only reflected in the behavior but also in the psychological perception, which, in turn, affects the performance and success of PPP projects. Therefore, it is necessary to take the public sector's attitude, that is, the relationship orientation, toward the private sector as the starting point to study its impact.

In PPP projects, the public sector pursues the maximization of social and public interests and the private sector pursues the maximization of their own interests (Zhang et al., 2019). This kind of natural opposition of project goals inevitably makes the two parties show opportunistic behavior in cooperation (Liu et al., 2016a,b; Li J. et al., 2018). In particular, as the executor of the project, the private sector will use the asymmetry of information to pursue its own interests and damage the interests of the public sector, resulting in an increase in project costs (including financing costs, communication costs, transaction costs, etc.) (Grout, 2003; Velotti et al., 2012; Šeba, 2015) and the reduction of project benefits (Li et al., 2005; Wang et al., 2020). Existing studies on opportunistic behavior of PPP projects mostly focus on building a game model for decision-making between public and private parties (Grout, 2003, Li X. et al., 2018) from the perspective of relationship governance and contract governance (Sabry, 2015) and qualitatively explore how to avoid opportunistic behavior of the private sector (Wang et al., 2018). There are few quantitative empirical studies.

In addition, more and more attention has been paid to the role of the private sector's justice perception in PPP partnership and project success (Wu et al., 2018). However, more attention has been paid to the impact of justice perception on relationship quality and project performance (Almarri and Blackwell, 2014; Du et al., 2017; Warsen et al., 2018) and few studies have explored the impact of justice perception on the private sector's opportunistic behavior.

The public sector is the main force to promote the healthy and standardized development of PPP projects, and its different relationship orientation has different effects on the private sector's JP and opportunistic behavior. However, there are few systematic analyses of relationship orientation of the public sector, and the mechanism of relationship orientation on the opportunistic behavior of the private sector is not clear. In order to explore the abovementioned research gaps, this study will design a measurement scale of the public sector's relationship orientation based on literature review, combined with PPP project practice and expert interview results, and propose an empirical model to explore the impact process of different public sector relationship orientations on the private sector's justice perception and opportunistic behavior in PPP projects.

Several potential theoretical contributions are included in this study. One is to propose the definition of the public sector's relationship orientation in PPP projects and summarize three dimensions [emotional relationship orientation (ERO), instrumental relationship orientation (IRO), and rent-seeking relationship orientation (RRO)]. On this basis, the questionnaire is designed and the measurement items are set according to the characteristics of PPP projects under the background of Chinese culture. The developed scale can be used for reference by other scholars and provide theoretical support for the follow-up research. The other is to analyze the relationship between the public sector's relationship orientation and the private sector's opportunistic behavior and explore the mediating role of justice perception. The research results will help to change the concept and attitude of the public sector, avoid the opportunistic behavior of the public sector, provide new research perspectives and ideas for related research, and facilitate the standardized and healthy development of PPP projects.

Therefore, the purpose of this study is to analyze the relationship among relationship orientation, justice perception, and opportunistic behavior. On the basis of the above discussion, the following two research questions (RQ) are addressed:

**RQ1:** How does the public sector's relationship orientation influence the private sector's opportunistic behavior?
**RQ2:** How does justice perception intervene between relationship orientation and opportunistic behavior?

## Theoretical Analysis and Hypothesis

### Theoretical Analysis

#### Relationship Orientation

Many Chinese scholars have studied the connotation of “relationship” and its socialization function. He and Qu (2006) first proposed the definition of “relationship orientation.” He believes that relationship orientation is the essence of Chinese relationship culture, and everything starts and ends with relationship. Zuo (2002) thinks that relationship orientation is not only an attitude but also a way of behavior and puts forward a specific definition of relationship orientation: “Relationship orientation is a kind of psychological tendency or behavior style. People use relationship as the basis for cognition of themselves or others, and then define themselves and make corresponding behavioral responses.” Existing research on relationship orientation mainly includes two aspects: one is to study the impact of individual-level relationship orientation on ethical behavior and relationship satisfaction (Law et al., 2000; Chen et al., 2001); the other is to study the influence of organizational-level relationship orientation on cooperation, knowledge sharing, organizational performance, etc. (Bock et al., 2005; Leung et al., 2014). Hwang classified the relationship orientation types at the individual level into three types: expressive relationship, instrumental relationship, and mixed relationship according to closeness and distance; Su and Littlefield (2001) divided the relationship orientation in Chinese business environment into “favor-seeking” relationship and “rent-seeking” relationship; Liu (2011) divided the relationship into three types: obligatory relationship, emotional relationship, and instrumental relationship when studying organizational relationships.

At present, the research on organizational relationship orientation mostly focused on business management, marketing, and other fields; less on project-based organizations; and much less on relationship orientation in engineering projects or PPP projects. The research of relationship in engineering project, such as project relationship governance, often focuses on the main factors such as communication, trust, and commitment between the two sides of the relationship. There is no research based on the attitude and behavior of the public sector, taking the relationship orientation of public sector as the research object, and exploring its mechanism of action. In PPP projects, the public sector is the main force to promote the project process, and its relationship orientation has a great impact on social capital in different stages of the project. The relationship orientation of the public sector is not static, but a dynamic process reflecting the influence of socialization. Different relationship orientations will show corresponding attitudes and actions, such as whether the public sector fully trusts the private sector, treats it sincerely and cooperates with each other as far as possible, or values the exchange of interests and mutual benefit, and whether the public sector will use its privileges to set obstacles for the private sector and obtain private benefits.

Referring to previous studies, this paper defines the public sector's relationship orientation in PPP projects as follows: at each stage of PPP projects, the public sector takes “relationship” as the basis for cognition of itself or the other party and defines its own role in the project on this basis, which is reflected by external attitude or corresponding behavior. The classification of relationship orientation refers to the component analysis of social relations of Chinese enterprises, and combined with project scenarios, the public sector's relationship orientation is divided into ERO, IRO, and RRO.

ERO means that in PPP projects, the public sector tends to establish a long-term friendly partnership with the private sector, emphasizes mutual emotional exchange, and shows a series of behavioral reactions such as sincerity, trust, mutual assistance, and support in cooperation; IRO is based on mutual benefit between the public sector and the private sector, emphasizing the unity of rights, responsibilities, and obligations, which is a rational relationship that restricts each other; rent-seeking relationship generally refers to the short-term exchange of power (political power or resource rights) and interests under the imperfect system. RRO in this paper refers to the attitude and behavior of the public sector to create rent actively or passively. The public sector or its representatives (individuals) use their part of franchise to obtain illegal property and rights from the private sector within the scope of power, resulting in a waste of social resources and a reduction in efficiency (Iossa and Martimort, 2016).

#### Opportunistic Behavior

Opportunism is a basic hypothesis in transaction cost economics. It was first proposed by Williamson and defined it as that in the case of asymmetric information, one party of a transaction misleads and deceives the other party to obtain private interests by not fully and truthfully disclosing information. Opportunism originally refers to the behavior that clearly violates the contract in the transaction. With the development of research, the behavior of destroying the relationship between the two sides of the transaction is also included in the meaning of opportunism. The research of Wathne and Heide (2000) divides opportunism into active opportunism and passive opportunism. Similarly, Luo classified opportunism behavior into strong opportunistic behavior (SOB) and weak opportunistic behavior (WOB) in the study of company transactions in emerging markets (Luo, 2006). Scholars have also carried out some discussions on opportunism in PPP projects. Research has found that in PPP projects, the conflict of interests of all participants will lead to opportunistic behavior (Lohmann and Rötzel, 2018, Wang and Zhang, 2019). Compared with the public sector, the private sector has the advantage of information and may be opportunistic and strive for greater benefits for itself (Falch and Henten, 2010). The opportunistic behavior of the private sector in PPP projects may seriously damage the public interest and affect the success of the project. Therefore, it is very important to understand and prevent the opportunistic behavior of the private sector (Wang and Zhang, 2019).

This paper defines the opportunistic behavior of the private sector in PPP projects as follows: During the life cycle of PPP projects, the private sector takes advantage of information to maximize its own interests at the cost of public sector interests (or public interests) by concealing or distorting information, withdrawing commitments, violating agreements or evading obligations. According to the common classification, this paper divides opportunistic behavior into SOB and WOB. SOB refers to the behavior that violates the terms or conditions clearly drafted in the contract; WOB refers to the behavior that violates the relationship norms, which is not recorded in the contract, but involves the common understanding of the public and private parties.

#### Justice Perception

The research on justice perception between organizations mostly focuses on the field of strategic alliance and supply chains, among which, represented by Luo et al. (2015), justice perception is divided into three dimensions: distributive justice, procedural justice, and interactive justice. In the research on the private sector's justice perception of PPP projects, Du et al. (2017) also drew lessons from Luo's viewpoint to divide the justice perception in dimensions. Referring to the above criteria, this paper divides private sector's justice perception into three dimensions: distributive justice (DJ), procedural justice (PJ), and interactive justice (IJ).

Justice perception in PPP projects is the subjective evaluation of the private sector's degree of satisfaction in the process of cooperation with the public sector. This subjective evaluation plays an important role in the behavior of the private sector and then affects the quality of the project relationship. DJ is based on Adams' equity theory, which emphasizes the equality of the payment and income distribution of all parties in the transaction (Yean and Yusof, 2016). DJ in PPP projects is mainly considered from two aspects of profit and risk. PJ initially refers to the fairness of the third party's adjudication procedure in dispute settlement (Aibinu et al., 2008). In PPP projects, it is mainly reflected in the rationality of the public sector's performance of duties and the effectiveness of the private sector's exercise of rights. With the further development of equity theory, Bies proposed the concept of IJ (Liu et al., 2012), which means the justice of mutual treatment and information exchange between border managers of all parties to the transaction. In PPP projects, the interaction between the two sides is also realized through the responsible people of each party, which is reflected in the mutual treatment and information exchange between the two parties.

### Hypothesis Development

#### The Influence of Relationship Orientation on Opportunistic Behavior

Research on attitude and behavior in organizational behavior has found that one party's attitude orientation will affect the other party's opportunistic behavior. Drawing on the relevant research results, the public sector's relationship orientation in PPP projects will also affect the private sector's opportunistic behavior. PPP projects have a long period. In the long-term cooperation between public and private parties, in addition to material exchanges, non-material social or emotional exchanges are also essential. ERO of public sector focuses on emotional exchange, which is mainly reflected in the sincerity and trust of the public sector to the private sector, and giving sufficient support and autonomy to the private sector.

If the private sector feels that the public sector trusts itself very much, provides support and help from the heart in the process of cooperation, and has sufficient autonomy during the project to give full play to its own advantages and strengths (Li X. et al., 2018), then the private sector will follow the contract as much as possible to reduce mutual suspicion and conflict; even if there is a conflict of interest, the private sector will be more inclined to believe that the public sector is well-intentioned and complies with emotion or morality, which will still reduce opportunistic tendency and behavior (including SOB and WOB). Therefore, ERO of public sector is a strong internal constraint mechanism for the private sector. The higher the level, the lower the possibility of opportunistic behavior in the private sector. Based on this, the following hypotheses are proposed:

**H1:** ERO negatively affects opportunistic behavior (**H1a:** ERO negatively affects SOB; **H1b:** ERO negatively affects WOB).

The core of exchanges between organizations is reciprocity, which may be the exchange of emotions or the exchange of economic benefits (Ngai et al., 2015). IRO of the public sector emphasizes the mutual benefit of interest exchange. There are two reasons for the public sector to provide assistance to the private sector: one is that the responsibilities, rights, and interests of both parties are clearly divided in the PPP contract, and the public sector needs to fulfill its obligations according to the contract requirements; on the other hand, it is expected that providing assistance can bring additional public benefits, such as improving the quality of products/services and achieving better environmental benefits. Once the feedback from the private sector does not meet expectations, the public sector will choose to terminate this assistance (Mofokeng and Chinomona, 2019).

Therefore, on the one hand, IRO can restrain SOB of the private sector through the mutual restriction of the contract; on the other hand, IRO has a certain utilitarian color, which is not conducive to the formation of stable and long-term partnership. The private sector may feel distrusted and choose to conceal some information in order to protect its own interests from being damaged, turn passive to active, and take more WOB. Therefore, the following assumptions are put forward:

**H2a:** IRO negatively affects SOB; **H2b:** IRO positively affects WOB.

Compared with ERO and IRO, the public sector, which is manifested as RRO, focuses on the short-term self-interest rather than the gain and loss of public interest. In PPP projects, the public sector mainly obtains private interests through part of franchise power and resources in its hands. For example, in the stage of contract negotiation, the public sector uses its strong position to formulate unfair risk sharing and benefit distribution clauses in the contract, and sets up unreasonable pricing and price adjustment mechanism; it is negligent in examination and approval or acceptance process and deliberately sets obstacles to the private sector; excessive intervention in the construction and operation process leads to the private sector not having corresponding autonomy. These behaviors of RRO will harm the interests of the private sector to a certain extent and aggravate the conflicts between the two sides (Carson et al., 2006). Then, the private sector is more likely to take opportunistic behaviors to make up for its own losses. In addition, when the private sector realizes that the public sector has a rent-seeking orientation, it will use bribery and other illegal or gray means to enter into transactions with the public sector, thereby fighting for competition protection or allowing the public sector to relax audit conditions. However, in China's social environment, people reject “exchange of power for money” and “power for personal gain” (Chen, 2007). Although this type of transaction will bring certain short-term benefits to the private sector, it will also cause psychological disgust and distrust in the private sector and then easily lead to opportunistic behavior. Therefore, this research proposes the following hypotheses:

**H3:** RRO positively affects opportunistic behavior (**H3a:** RRO positively affects SOB; **H3b:** RRO positively affects WOB).

#### The Influence of Relationship Orientation on Justice Perception

In the general organization or business cooperation, ERO is often based on the feelings and trust established by the historical transaction experience with the partners, but in the temporary organization such as PPP projects, ERO of the public sector is based on its initial relationship with the private sector. The establishment of this initial relationship is the result of comprehensive consideration of the private sector's past project experience, executive ability, qualification, and reputation (Li, 2019). Under the high expectation of the private sector, the public sector will show an ERO. Specifically reflected in the subsequent contract negotiation, the public sector will set corresponding contract terms according to the expected behavior of the private sector in the performance of the contract (Abdullah and Khadaroo, 2020), so as to protect the rights and interests of the private sector. In this way, ERO will be transformed into the rationality of risk sharing and benefit sharing in the contract and the consistency of action, which will bring a certain degree of fairness to the private sector.

Therefore, the public sector's ERO will affect the design of PPP project contract terms and thus affect the private sector's justice perception, including DJ and PJ. In addition, after the signing of the contract, ERO will increase the emotional connection and communication between the two sides, respect each other, jointly solve the problems in the project (Liu and Wang, 2016), and improve the level of IJ of the private sector. Therefore, the following hypotheses are put forward:

**H4:** ERO positively affects justice perception (**H4a:** ERO positively affects DJ; **H4b:** ERO positively affects PJ; **H4c:** ERO positively affects IJ).

IRO emphasizes the mutual restriction between the public and private parties and shows the characteristics of “instrumental rationality” in the cooperative. On the one hand, it shows the contract spirit or consciousness of the public sector. The public sector is aware that it is in an equal subject position with the private sector that has concluded the contract. This is reflected in the action of conscientiously fulfilling the responsibilities and obligations stipulated in the contract, such as risk sharing, payment on time, timely price adjustment, etc. These behaviors will ensure a higher justice perception of the private sector. On the other hand, the premise of IRO public sector to extend a helping hand to the private sector is that through rational analysis and calculation, considerable future benefits are expected, and the two parties are in a dynamic game relationship. Under this mentality, in order to allow the private sector to provide better products/services, the public sector will also make appropriate concessions or compromises. The interaction between the two parties is harmonious and equal, which is conducive to achieving reasonable returns of the project. Although the private sector has not achieved the goal of high rate of return, it has ensured long-term and stable returns, reduced risks, and helped to achieve equity. Therefore, the following assumptions are put forward:

**H5:** IRO positively affects justice perception (**H5a:** IRO positively affects DJ; **H5b:** IRO positively affects PJ; **H5c:** IRO positively affects IJ).

The rent-seeking relationship between the public sector and the private sector adheres to the principle of “no profit, no gain.” The premise and motivation for action are whether the privilege can be used to obtain personal economic benefits. In cooperation with the private sector, risks that do not match its capabilities may be transferred to the private sector, ignoring the interests of the private sector, or using coercive rights to interfere with the process of the project, or being “superior” and uncooperative in the communication process. Out of the need for resources, the private sector often has to assess the situation and compromise the rent-seeking behavior of the public sector (Lim and Loosemore, 2017), and it is difficult to feel fair. Therefore, RRO is not conducive to the formation of private sector's justice perception, and the following hypotheses are proposed:

**H6:** RRO negatively affects justice perception (**H6a:** RRO negatively affects DJ; **H6b:** RRO negatively affects PJ; **H6c:** RRO negatively affects IJ).

#### The Influence of Justice Perception on Opportunistic Behavior

Justice perception is a powerful predictor of organizational behavior response. Current studies have found the importance of justice perception in the cooperation between the public sector and the private sector (Gerlach, 2019, Zhang and Jia, 2010). The degree of justice perception in the private sector can be used to predict whether the two sides will take actions to destroy the cooperative relationship in cooperation. Different from the relatively equal market position of both parties in the supply chain and strategic alliance, in the actual operation of PPP projects in China, the public sector is often in an absolutely strong position in the transaction relationship. In addition, the legislation on PPP projects is not yet perfect, and there is a lack of constraints on the public sector, which makes it easy to place the private sector in an unfair situation. When the private sector has a low perception of justice, it will adopt opportunistic and other uncooperative tendencies or behaviors, such as frequently creating disputes during the project process (Xiong et al., 2018), trying to achieve the fair state recognized by itself through renegotiation or secondary operations, and using information asymmetry to retaliate against the public sector's unfairness to oneself by deliberately concealing and distorting key project information, leading to intensified conflicts in the project. On the contrary, when the degree of justice perception is high, the value and advantages of the private sector in the project can be reflected, bringing a sense of respect and belonging (Ellis et al., 2009), which makes the private sector more willing to abide by the contract, actively cooperate with the public sector in cooperation, and conduct open communication, thereby reducing opportunistic behavior. Therefore, this study considers that a higher level of justice perception can effectively reduce opportunistic behavior and puts forward the following hypotheses:

**H7:** DJ negatively affects opportunistic behavior (**H7a:** DJ negatively affects SOB; **H7b:** DJ negatively affects WOB).
**H8:** PJ negatively affects opportunistic behavior (**H8a:** PJ negatively affects SOB; **H8b:** PJ negatively affects WOB).
**H9:** IJ negatively affects opportunistic behavior (**H9a:** IJ negatively affects SOB; **H9b:** IJ negatively affects WOB).

**Figure 1:**
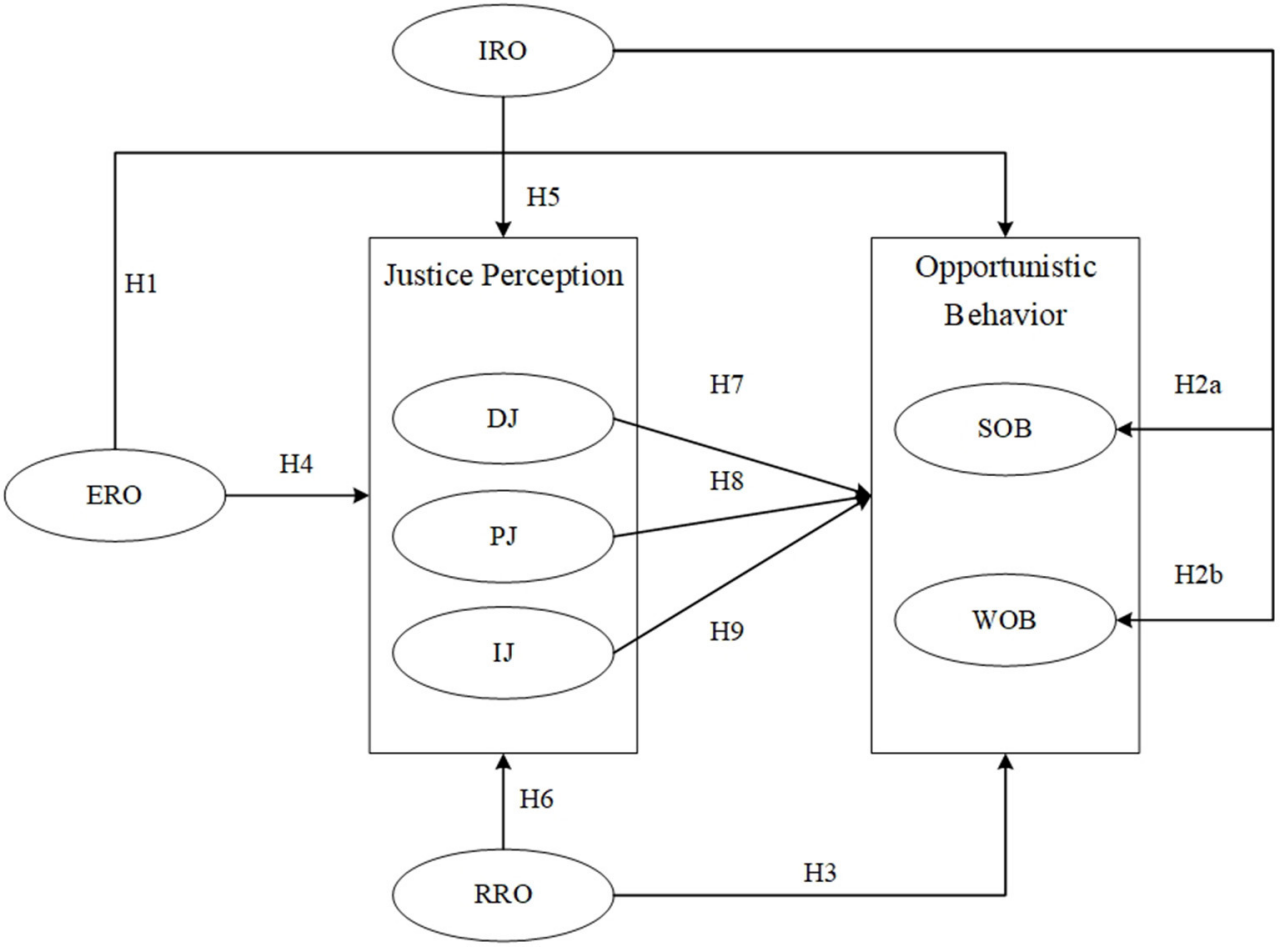
Conceptual model of the effect of public sector's relationship orientation.

Based on the above hypotheses, a conceptual model of the impact of public sector's relationship orientation on private sector's justice perception and opportunistic behavior is proposed, as shown in Figure 1.

## Methodology

### Research Approach

This study adopts an empirical research method, which is mainly divided into two parts. One is to collect data through questionnaire survey. The concepts involved in this study include the public sector's relationship orientation and the private sector's justice perception and opportunistic behavior. These variables are potential variables, and their data cannot be obtained from open sources such as open databases. Therefore, questionnaire survey is needed to collect data. In addition, questionnaire survey is a commonly used method of collecting data in empirical research, and its effectiveness is widely recognized (Faiz et al., 2019). The second is to organize and analyze the collected data through statistical analysis software and use the structural equation model (SEM) method to verify the proposed hypothesis and model. In the empirical research stage, considering that relationship orientation, justice perception, and opportunistic behavior of the PPP projects are complex and difficult to measure directly, and because the proposed model is complex, SEM is selected to test the adaptability of the model and the correctness of the hypothesis. SEM integrates factor analysis and interaction path analysis between variables, which can be used to verify complex phenomena, so as to quantify practical problems and better understand the nature of problems.

### Questionnaire Development

In order to ensure the validity and rationality of the questionnaire designed on the basis of previous research results, this paper follows the following steps to form the final questionnaire: firstly, through literature induction, combining the relevant research, on the basis of fully understanding the connotation of each variable, combining the variable scale proposed by predecessors with PPP project management background and Chinese practice, analyzing its accuracy and rationality, strictly selecting the measurement items of each variable, and making reasonable adjustments to prepare the first draft of the questionnaire. Secondly, invite a group of PPP project management research experts and potential interviewees to interview, and revise the initial questionnaire accordingly. Finally, to ensure the validity and rationality of each measurement item, discuss the measurement items one by one with the academic team teachers and doctoral students to form the final questionnaire.

### Sampling and Data Collection

The questionnaire of this study is distributed to the private sector, which is mainly composed of managers in large construction enterprises who participate in PPP project practice, financial institutions and investors involved in PPP project investment, and scholars with practical experience in PPP projects, covering municipal public utilities, transportation, urban comprehensive development, environmental protection, energy and power, and other fields.

The questionnaires were collected by e-mail and on-site distribution. A total of 325 questionnaires were distributed, 297 questionnaires were returned, and 209 were valid questionnaires. The effective rate of the questionnaire was 70.9%, which met the model test conditions. From the sample distribution, 65.07% of the respondents are middle and senior managers, 89.95% are undergraduates or above, and 62.68% of respondents have been engaged in PPP projects for more than 3 years. The respondents have a high degree of understanding the entire process of PPP project, which ensures the validity of the survey data. Table 1 displays the statistical results of respondents. Before using SEM analysis, SPSS 22.0 was used to do KMO and Bartlett sphere test on sample data. The results showed that KMO values of research variables were all >0.8, and the statistical values reached a significant level, which proved that it was suitable for factor analysis.

**Table 1:** Sample characteristics.

**Measure**	**Items**	**Frequency**	**Percentage (%)**
Industry type	Municipal engineering	66	31.58
	Transportation	51	24.40
	Urban comprehensive development	34	16.27
	Ecological construction and environmental protection	22	10.53
	Energy power	19	9.09
	Others	17	8.13
Education	Ph.D. and above	23	11.00
	Master	90	43.06
	Graduate	75	35.89
	Undergraduate	21	10.05
Work experience	Less than 3 years	78	37.32
	3–5 years	91	43.54
	6–8 years	28	13.40
	More than 8 years	12	5.74
Position	Senior management	26	12.44
	Department Manager/Deputy Manager	49	23.44
	Professional director	61	29.19
	General staff	73	34.93
Work unit type	Local state-owned enterprises	85	40.67
	Central enterprises and their subsidiaries	77	36.84
	Private enterprise	24	11.48
	Foreign enterprise	15	7.18
	Others	8	3.83

### Measurement

At present, there are few measurement scales for the independent variable: “public sector's relationship orientation” of PPP projects. This paper draws on the relevant research of strategic alliance and marketing enterprises, as well as the research on PPP project governance by Wang S.Q. and Du Y.L. in China. Based on the connotation and definition of relationship orientation, this paper divides the relationship orientation into three dimensions: ERO, IRO, and RRO, each including five measurement items. The research on dependent variable: “opportunism behavior” is relatively mature. Referring to the research of Luo et al., this paper divides opportunistic behavior into SOB and WOB. The measurement scale integrates the characteristics of the PPP project and designs nine measurement items. Among them, four are SOB and five are WOB. The research on the private sector's justice perception is relatively perfect in PPP projects, and the scale is relatively mature. This paper refers to the research and scale of Du Y.L. and Sun N. The three kinds of justice perceptions are measured by four items respectively.

All survey items are listed in Table 2. All items in all scales were measured on a five-point Likert scale, from 1 (strongly disagree) to 5 (strongly agree). Likert-type scales are effective for subjective questions that aim to measure subjective states, such as opinions, knowledge, feelings, and perceptions. Cronbach's alpha for relationship orientation, justice perception, and opportunistic behavior are shown in Table 3.

**Table 2:** List of coded survey items.

**Variable**	**Code**	**Scale item**
Emotional relationship orientation	ERO1	The public sector is willing to provide assistance when we need it
	ERO2	The public sector can treat us sincerely
	ERO3	The public sector is willing to share their feelings and experiences of cooperation with us
	ERO4	We can understand and negotiate with each other when there are objections in cooperation
	ERO5	The public sector believes that we have the ability to achieve the expected goals and provide support
Instrumental relationship orientation	IRO1	The public sector interacts with us mainly to achieve its expected goals (such as the social benefits, environmental benefits, public satisfaction of PPP projects, etc.)
	IRO2	The communication between the public sector and us follows the principle of “Business is business”
	IRO3	Unless it is helpful to achieve project success or improve project performance, the public sector will not provide us with help or support other than contractually agreed
	IRO4	The degree of assistance provided by the public sector to us is often determined by the improvement of our project performance
	IRO5	The public sector provides help for us in order to allow us to provide better products or services
Rent-seeking relationship orientation	RRO1	The public sector is in a strong position in contract negotiations and operates according to its own will (such as unreasonable risk sharing, excessive intervention in the operation management process, etc.)
	RRO2	The public sector uses administrative power to set up barriers to provide competition protection for certain private sectors
	RRO3	The public sector uses departmental legislation to form monopoly prices, leading to excessive pricing or price adjustment mechanisms that are not in line with reality
	RRO4	The public sector sometimes formulates cumbersome administrative examination and approval procedures and deliberately set up obstacles
	RRO5	The public sector sometimes relaxes the review standards in the process of examination and supervision
Strong opportunistic behavior	SOB1	Sometimes we do not invest resources (such as funds, human resources, equipment or materials) as required by the contract
	SOB2	Sometimes we do not provide accurate key information to the public sector in accordance with the contract
	SOB3	When there is a dispute or conflict in the implementation of the project, sometimes we do not fully follow the procedures agreed in the contract
	SOB4	Sometimes we will not comply with the agreement with the public sector for our own benefit
Weak opportunistic behavior	WOB1	Sometimes we will provide slightly untrue information to the public sector for our own benefit
	WOB2	Sometimes we will conceal something from the public sector for our own benefit
	WOB3	Sometimes we will make promises to the public sector that may not be fulfilled
	WOB4	Sometimes we will use loopholes in the agreement with the public sector to seek benefits
	WOB5	Sometimes we don't do our best in partnership with the public sector
Distributive justice	DJ1	Compared with similar projects, our (private sector) rewards from the project are fair
	DJ2	Compared with similar projects, our (private sector) resource allocation/preferential policies obtained from the project are fair
	DJ3	Our (private sector) returns from the project match the resources and efforts we put in
	DJ4	Our (private sector) returns from the project match the level of performance achieved
Procedural justice	PJ1	The establishment of the PPP project transaction structure and decision-making procedures are fair
	PJ2	The procedures of PPP contract terms signing and contract negotiation are fair
	PJ3	The procedures for the management and supervision of PPP contracts by the public sector are fair
	PJ4	The procedures for PPP projects to allocate risks and benefits are fair, transparent and consistent
Interactive justice	IJ1	The public sector is sincere and frank in solving problems, providing the real information needed by the project
	IJ2	When the public sector interacts with us (the private sector), respect our opinions from a win-win perspective
	IJ3	Both parties of the project can always provide timely and accurate feedback to each other as much as possible
	IJ4	Both parties to the project work together to conduct open and direct communication

**Table 3:** Reliability and convergent validity analysis results.

**Reliability analysis**					**Convergent validity analysis**			
**Variable**	**Code**	**CITC**	**α after deleting this item**	**Cronbach's α**	**Standardization factor load**	**AVE**	**CR**	**Judgment index**
ERO	ERO1	0.756	0.815	0.841	0.784	0.527	0.854	χ2/df = 1.462RMSEA = 0.038RMR = 0.032TLI = 0.984NFI = 0.941GFI = 0.952CFI = 0.975IFI = 0.982AGFI = 0.922
	ERO2	0.784	0.823		0.753			
	ERO3	0.643	0.835		0.746			
	ERO4	0.736	0.826		0.751			
	ERO5	0.622	0.814		0.794			
IRO	IRO1	0.719	0.810	0.813	0.719	0.561	0.861	
	IRO2	0.660	0.803		0.721			
	IRO3	0.764	0.794		0.751			
	IRO4	0.716	0.786		0.718			
	IRO5	0.694	0.809		0.794			
RRO	RRO1	0.742	0.815	0.832	0.735	0.573	0.872	
	RRO2	0.798	0.830		0.781			
	RRO3	0.643	0.819		0.768			
	RRO4	0.754	0.824		0.754			
	RRO5	0.683	0.803		0.781			
DJ	DJ1	0.741	0.831	0.872	0.821	0.548	0.867	
	DJ2	0.762	0.852		0.719			
	DJ3	0.768	0.844		0.727			
	DJ4	0.781	0.863		0.745			
PJ	PJ1	0.765	0.843	0.867	0.753	0.594	0.857	
	PJ2	0.781	0.827		0.786			
	PJ3	0.763	0.859		0.767			
	PJ4	0.742	0.845		0.766			
IJ	IJ1	0.751	0.853	0.878	0.759	0.523	0.843	
	IJ2	0.768	0.869		0.767			
	IJ3	0.767	0.872		0.772			
	IJ4	0.759	0.859		0.764			
SOB	SOB1	0.712	0.894	0.942	0.794	0.575	0.891	
	SOB2	0.694	0.885		0.788			
	SOB3	0.738	0.891		0.767			
	SOB4	0.772	0.912		0.776			
WOB	WOB1	0.684	0.893	0.921	0.768	0.566	0.882	
	WOB2	0.793	0.904		0.791			
	WOB3	0.786	0.871		0.795			
	WOB4	0.746	0.887		0.776			
	WOB5	0.715	0.894		0.763			

## Results and Analysis

### Reliability Analysis

In this study, SPSS 22.0 software was used to analyze the reliability of all latent variables. The results are shown in Table 3. The CITC values of all items were >0.50, indicating that the scale has high reliability. Cronbach's α values were significantly higher than 0.8 (the minimum was 0.807). After deleting this item, the α values of Cronbach's α were less than those of the original scale, indicating that the model scale has good reliability and high internal consistency.

### Common Methods Variance

In order to avoid homologous variance, neutral context was used in the design of the questionnaire. SPSS 22.0 statistical software was used to test the homologous variance, and all variables were processed centrally. The results of collinearity test of all variables showed that the VIF was lower than 10 (the maximum was 1.964), and the maximum tolerance was 0.784 (all <1); that is, the homologous variance was not significant and in a controllable state.

### Validity Analysis

The validity test is divided into two aspects: convergent validity and discriminant validity. Confirmatory factor analysis (CFA) was performed by AMOS 24.0 software to test the fitting of the model. The criteria of fitting degree included χ^2^/df <2; RMSEA <0.05; RMR <0.05; TLI, NFI, GFI, CFI, and IFI were all >0.9; and AGFI was close to 0.9. It can be seen from Table 1 that the load values of standardization factors of all latent variables were >0.7, which had a good fitting degree. The AVE values of the average variance extraction amount of each factor were >0.5, and the CR values were greater than the critical value of 0.7, indicating that the model had good convergent validity.

Discriminant validity used SPSS 20.0 software to obtain the correlation coefficient of each variable. As shown in Table 4, the diagonal bold part of the table was the square root of AVE, and the correlation coefficients of each variable and other variables were less than the corresponding square root of the AVE value, indicating that the scale had high discriminant validity.

**Table 4:** Discriminant validity analysis.

**Variable**	**1**	**2**	**3**	**4**	**5**	**6**	**7**	**8**
1 ERO	**0.726**							
2 IRO	0.524	**0.749**						
3 RRO	0.657	0.443	**0.757**					
4 DJ	0.527	0.612	0.387	**0.740**				
5 PJ	0.533	0.628	0.446	0.587	**0.771**			
6 IJ	0.581	0.534	0.527	0.549	0.566	**0.723**		
7 SOB	0.627	0.567	0.511	0.548	0.622	0.535	**0.758**	
8 WOB	0.552	0.594	0.539	0.621	0.634	0.524	0.529	**0.752**

*The diagonal bold values are the square root of AVE*.

### Hypotheses Testing and Discussion

According to the initial SEM constructed above, the path analysis is carried out using Amos 24.0 software and the results are shown in Table 5.

**Table 5:** Results of hypothesis test.

**Action path**	**Standardized path coefficient**	***P*-value**	**Test results**
H1a: ERO → SOB	−0.254	^*^	Passed
H1b: ERO → WOB	−0.213	^*^	Passed
H2a: IRO → SOB	−0.443	^***^	Passed
H2b: IRO → WOB	−0.351	^**^	Refused
H3a: RRO → SOB	0.242	^*^	Passed
H3b: RRO → WOB	0.305	^**^	Passed
H4a: ERO → DJ	0.213	^*^	Passed
H4b: ERO → PJ	0.083	0.314	Refused
H4c: ERO → IJ	0.149	^*^	Passed
H5a: IRO → DJ	0.359	^**^	Passed
H5b: IRO → PJ	0.401	^***^	Passed
H5c: IRO → IJ	0.249	^**^	Passed
H6a: RRO → DJ	−0.394	^***^	Passed
H6b: RRO → PJ	−0.427	^***^	Passed
H6c: RRO → IJ	−0.308	^**^	Passed
H7a: DJ → SOB	−0.346	^***^	Passed
H7b: DJ → WOB	−0.322	^**^	Passed
H8a: PJ → SOB	−0.504	^***^	Passed
H8b: PJ → WOB	−0.416	^***^	Passed
H9a: IJ → SOB	−0.287	^*^	Passed
H9b: IJ → WOB	−0.318	^**^	Passed

* *p < 0.05*,

** *p < 0.01*,

*** *p < 0.001*.

#### The Effect of Relationship Orientation on Opportunistic Behavior

Suppose that the *P*-values of H1a and H1b are both significant, and H1 is verified. Assuming that the path coefficient of H2a is −0.443 and the *P*-value is significant at the level of 0.001, the verification is passed; assuming that the path coefficient of H2b is −0.351, the original hypothesis that IRO has a positive impact on WOB has not been verified, but the *P*-value is significant at the level of 0.01, which indicates that IRO has a negative impact on WOB.

This may be because on the one hand, under the IRO of the public sector, the two partners adhere to the “business is business” style, which makes the two sides in a state of equal balance of rights, responsibilities, and interests, and the WOB will also weaken; on the other hand, under the Chinese characteristics, the private sector is dominated by state-owned enterprises, and most of the respondents in this study are from state-owned enterprises (the proportion is 77.51%, of which central enterprises and their subordinate companies accounted for 36.84%, and the local state-owned enterprises accounted for 40.67%). In PPP projects, the state-owned enterprises, which represent the private sector, pay more attention to the maintenance of reputation and brand, and are committed to long-term cooperation with the public sector, reflecting the sense of social responsibility of state-owned enterprises, so they tend not to take WOB. Suppose that the *P*-values of H3a and H3b are significant, and that H3 is verified.

#### The Effect of Relationship Orientation on Justice Perception

Assuming that the *P*-values of H4a and H4c are both significant, the verification is passed; assume that the path coefficient of H4b ERO on PJ is 0.083, but *P*-value is 0.314 > 0.05, the hypothesis is rejected. This shows that the ERO has no significant effect on the PJ perception of private sector, which may be due to the fact that the public sector's ERO shows mutual sincere trust with the private sector, and there are some cases in which the contract procedure is not fully implemented in the process of cooperation.

Some interviewees who received the return interview gave examples of this situation: For example, for projects with a tight schedule during the construction phase, based on the good qualification and performance of the private sector, the public sector requires the construction unit to enter the site in advance to organize and prepare for the project before signing the contract; after the PPP project is completed, based on the long-term and friendly cooperation between the two parties, the private sector communicates and coordinates with the public sector, and the public sector agreed to shorten the acceptance period or reset the time node of the operation period, so that the project can enter the operation period and repurchase period ahead of schedule. Although this situation is not strictly implemented in accordance with the requirements of the contract, it also ensures that both parties reach an agreement. In addition, after several years of development, many PPP projects in China have entered the operation period. Processes such as PPP project decision-making, contract signing and negotiation, and profit and risk allocation procedures, which reflect PJ, are less involved in the project operation period, so the ERO will also be affected, and the direct effect on PJ is not significant. It is assumed that H5a, H5b, H5c, H6a, H6b, and H6c all pass the validation.

#### The Effect of Justice Perception

**The direct effect of justice perception on opportunistic behavior**
It is assumed that the path coefficients of H7a, H7b, H8a, H8b, H9a, and H9c are consistent with the assumed direction, and the *P*-values are all significant. The hypothesis is verified.
**The mediating effect of justice perception in the influence of relationship orientation on opportunistic behavior**
The mediating effect of justice perception is tested by SEM based on Bootstrapping and verified by Smart PLS statistical software. The results are shown in Table 6. Among them, the total indirect effect of the public sector's ERO on opportunistic behavior is −0.191, which is significant at the level of 0.05; the total indirect effect of IRO on opportunistic behavior is −0.245, which is significant at the level of 0.01; the total indirect effect of RRO on opportunistic behavior is 0.207, which is significant at the level of 0.05. It shows that there is a mediating effect between relationship orientation and opportunistic behavior. When the mediating variable “justice perception” is introduced, the path coefficients of antecedent variable ERO to the dependent variable opportunism behavior are −0.133, −0.187, and 0.161, with *P*-values of *P* < 0.01, *P* < 0.001, and *P* < 0.01. It indicates that the intermediary relationship is established and justice perception plays a part of mediating role between public sector's relationship orientation and private sector's opportunistic behavior.

**Table 6:** Test results of mediating effect.

**Hypothetical path**	**Total indirect effects: c–c^**′**^**	**Indirect effect: ab**	***P*-value**	**95% confidence interval**	
				**lower limit**	**upper limit**
ERO → opportunistic behavior	−0.191		^*^	0.164	0.327
ERO → justice perception → opportunistic behavior		−0.133	^**^	0.121	0.275
IRO → opportunistic behavior	−0.245		^**^	0.043	0.237
IRO → justice perception → opportunistic behavior		−0.187	^***^	0.104	0.258
RRO → opportunistic behavior	0.207		^*^	0.186	0.394
RRO → justice perception → opportunistic behavior		0.161	^**^	0.117	0.249

* *p < 0.05*,

** *p < 0.01*,

*** *p < 0.001*.

## Conclusions

This study explores the mechanism of the public sector's relationship orientation on the private sector's justice perception and opportunistic behavior in PPP projects. Through literature review and theoretical analysis, this paper puts forward the connotation of the public sector's relationship orientation, and divides it into three types: ERO, IRO, and RRO. Combined with PPP project practice and expert interviews, this paper develops the measurement scale of the public sector's relationship orientation in PPP project and then puts forward the hypothesis to construct a SEM of relationship orientation, justice perception, and opportunistic behavior. In the verification of the model, AMOS software is used to verify the direct effect of each variable, and Smart PLS software is used to verify the mediating effect of justice perception. Draw the following research conclusions.

First, the public sector's relationship orientation has a significant effect on the formation and development of the private sector's justice perception and the emergence of opportunistic behavior. In PPP projects, one of the most important performance of the public sector's ERO is to pay attention to the cultivation and maintenance of long-term and stable partnership between the two sides. In the process of cooperation with the private sector, the private sector is given full trust and support, so that the private sector can feel enough trust and respect, and the sense of justice will also be enhanced. The natural differences between the two sides will also be more integrated due to sincere and frank communication and negotiation with each other, so as to minimize conflicts and disputes between the two sides. In this cooperative atmosphere, the advantages of private sector in management and technology can be better displayed, so as to reduce the opportunistic tendency and behavior.

The impact of IRO is mainly reflected in the signing and implementation of PPP project agreements. Driven by the IRO, the public sector will better serve its own role. From the initial stage of the project, it will rationally position and divide the interest boundary of both sides, and complete the task with due diligence. The cooperation between the private sector and the public sector is in a relatively equal game process. Both sides reach an agreement through equal negotiation, risk sharing, and benefit sharing, which ensures the reasonable return of the private sector, thus improving its justice perception, and helping it adjust the self-interest attribute of pursuing maximum interests, and reduce opportunistic behavior from the perspective of win–win. RRO is easy to occur in the case of the non-standard PPP project management system. The public nature of PPP projects and the scarcity of project resources endow the public sector with natural privileges, such as the right to choose, decide, and supervise. This makes the public sector always in a strong position in the project. Active or passive use of the privilege produces rent-seeking behavior. On the one hand, it will transfer too much risk to the private sector, which will damage the equal and stable partnership and cause a strong sense of unfairness in the private sector. On the other hand, in order to make up for the loss of its own interests, the private sector will use information advantages to break the contract, resulting in the products/services provided not meeting the public requirements. Therefore, in PPP projects, the different relationship orientations of the public sector have different influence mechanisms on the private sector's justice perception and opportunistic behavior, and there are great differences. ERO and IRO play a positive role, which is conducive to the good development of the project, while RRO is the opposite.

Second, justice perception plays a mediating role in the process of the public sector's relationship orientation acting on the private sector's opportunistic behavior. In the empirical study, the direct effect of justice perception on opportunistic behavior has been verified, which is consistent with the previous scholars' conclusions on the effect of justice perception on cooperative relationship in PPP projects. A good sense of justice is helpful to restrain the opportunistic behavior of the private sector, reduce conflicts and disputes between the public and private sectors, and improve the quality of the partnership. In addition, Smart PLS statistical software is used to prove the mediating role of justice perception in the influence of relationship orientation on opportunistic behavior. The verification of mediating effect further explains the internal mechanism of relationship orientation on opportunistic behavior: on the one hand, the public sector's relationship orientation directly affects the private sector's opportunistic behavior; on the other hand, it has an indirect impact through the private sector's justice perception. In the practice of PPP projects, the relationship orientation of the public sector will gradually form a stable relationship connection or a clear contract spirit in the cooperation with the private sector, which will affect the formation and development of the private sector's justice perception and then affect the opportunistic behavior.

Third, IRO is more conducive to reduce the private sector's opportunistic behavior. The empirical results show that from the perspective of different relationship orientations of the public sector, RRO will increase the opportunistic behavior of the private sector; IRO will not enhance WOB of the private sector, but will have a strong inhibitory effect on SOB and WOB; ERO will also weaken the opportunistic behavior. In addition, from the path coefficient and significance of ERO and IRO, the effect of IRO on reducing opportunistic behavior is more significant. This shows that the stronger the “rational” IRO in the public sector (for example, the clearer the public and private parties' cognition of their own roles and status, the more reasonable the project goals set, the more appropriate use of their own advantages, and the more firm the concept of pursuing win–win development), compared with the “emotional” ERO, which emphasizes mutual emotional support, the more conducive to avoiding discordant factors such as opportunistic behavior and the more conducive to the constant standardization and maturity of PPP projects and the improvement of cooperation efficiency.

## Practical Implications, Limitations, and Future Research

### Practical Implications

First, the public sector should accelerate the transformation of concept and find a reasonable role in PPP projects. In the current PPP system, it is difficult for the public sector to regard the private sector as the subject of equal cooperation in PPP projects and cannot strictly follow the basic principles of risk and benefit sharing, which is not conducive to the construction of public partnership system.

With the healthy development of the PPP model in China, the public sector should improve the ability to grasp PPP projects, fully understand the existing laws and policies, be good at using expert advice in the project start-up stage, clear their own target positioning, set reasonable and legal project structure and agreement arrangement for PPP projects, and try to avoid the destruction of their “strong gene” on the partnership, which is conducive to the development of the project. In cooperation with the private sector, the public sector and the private sector should treat each other honestly and communicate openly to reduce unnecessary frictions and disputes; at the same time, they should pay more attention to the “spirit of contract” and restrict their own behavior, so as to achieve the win–win goal of sharing risks and reasonable returns.

Second, further improve the top-level design of PPP projects and standardize the use of public sector privileges. The scarcity of resources and public attributes of PPP projects endow the public sector with the privilege of selection, supervision, and decision-making. This natural advantage brings the opportunity of rent-seeking for the public sector under the imperfect system. Therefore, in the future PPP legislation process, firstly, the division of responsibilities of each executive department can be further clarified, the responsibilities and obligations of the public sector in different stages of the project can be subdivided, and individual responsibility can even be emphasized, rather than merely stay on the accountability of departments or enterprises; the second is to improve the system construction. A cross-departmental PPP management agency can be established to professionally be responsible for the approval and supervision of PPP projects; finally, improve the public participation mechanism, give full play to public power and strengthen public supervision. In the whole process of the PPP project, information should be open and transparent, and supervision channels should be unblocked, thereby restricting the privileges of the public sector to a certain extent, enabling the public sector to avoid rent-seeking relations as much as possible, reducing rent-seeking behavior, and improving the utilization efficiency of public resources.

### Limitations and Future Research Directions

This study proposes the connotation and dimensions of the public sector's relationship orientation in PPP projects and explores its impact on the private sector through empirical research. The conclusion is creative, but there are also limitations, which can be further improved and discussed in future research. First, the relationship orientation will affect the project governance mechanism and then affect the project performance. Future research can start from the perspective of project governance to study the mechanism of relationship orientation. Second, in Chinese current PPP projects, the role of the private sector is still played by state-owned enterprises to a large extent. Therefore, data collection will have certain limitations. In the future, we can further explore the effect of the public sector's relationship orientation on the private sector in the private sector. Third, the current data are only from China, and the respondents are the private sector. In the future, comparative studies can be carried out. On the one hand, it is possible to compare whether the data of the public sector and private sector in PPP projects will have different results; on the other hand, we can collect data from other countries and regions for comparison, so as to further enrich and improve the research conclusions.

## Data Availability Statement

The raw data supporting the conclusions of this article will be made available by the authors, without undue reservation.

## Ethics Statement

Written informed consent was obtained from the individual(s) for the publication of any potentially identifiable images or data included in this article.

## Author Contributions

GF contributed to the research design, literature review, methodology, interview data collection and analysis, and original draft preparation. XL revised the methodology and reviewed and edited the manuscript. SH had supervisory roles and contributed to problem analysis. All authors contributed to the article and approved the submitted version.

## Conflict of Interest

XL was employed by the company China International Economic Consultants. The remaining authors declare that the research was conducted in the absence of any commercial or financial relationships that could be construed as a potential conflict of interest.
